# Vaccination strategies for future influenza pandemics: a severity-based cost effectiveness analysis

**DOI:** 10.1186/1471-2334-13-81

**Published:** 2013-02-11

**Authors:** Joel K Kelso, Nilimesh Halder, George J Milne

**Affiliations:** 1 School of Computer Science and Software Engineering, University of Western Australia, Stirling Highway, Crawley, Western Australia 6009, Australia

**Keywords:** Pandemic influenza, Cost effectiveness, Vaccination, Antiviral medication, Social distancing

## Abstract

**Background:**

A critical issue in planning pandemic influenza mitigation strategies is the delay between the arrival of the pandemic in a community and the availability of an effective vaccine. The likely scenario, born out in the 2009 pandemic, is that a newly emerged influenza pandemic will have spread to most parts of the world before a vaccine matched to the pandemic strain is produced. For a severe pandemic, additional rapidly activated intervention measures will be required if high mortality rates are to be avoided.

**Methods:**

A simulation modelling study was conducted to examine the effectiveness and cost effectiveness of plausible combinations of social distancing, antiviral and vaccination interventions, assuming a delay of 6-months between arrival of an influenza pandemic and first availability of a vaccine. Three different pandemic scenarios were examined; mild, moderate and extreme, based on estimates of transmissibility and pathogenicity of the 2009, 1957 and 1918 influenza pandemics respectively. A range of different durations of social distancing were examined, and the sensitivity of the results to variation in the vaccination delay, ranging from 2 to 6 months, was analysed.

**Results:**

Vaccination-only strategies were not cost effective for any pandemic scenario, saving few lives and incurring substantial vaccination costs. Vaccination coupled with long duration social distancing, antiviral treatment and antiviral prophylaxis was cost effective for moderate pandemics and extreme pandemics, where it saved lives while simultaneously reducing the total pandemic cost. Combined social distancing and antiviral interventions without vaccination were significantly less effective, since without vaccination a resurgence in case numbers occurred as soon as social distancing interventions were relaxed. When social distancing interventions were continued until at least the start of the vaccination campaign, attack rates and total costs were significantly lower, and increased rates of vaccination further improved effectiveness and cost effectiveness.

**Conclusions:**

The effectiveness and cost effectiveness consequences of the time-critical interplay of pandemic dynamics, vaccine availability and intervention timing has been quantified. For moderate and extreme pandemics, vaccination combined with rapidly activated antiviral and social distancing interventions of sufficient duration is cost effective from the perspective of life years saved.

## Background

The emergence of a new influenza strain to which the global population has little or no immunity is a continuing threat to human health. In the past century such influenza strains have caused pandemics that have ranged in severity from what might be termed mild – being little more pathogenic than seasonal influenza, as in the case of the 2009 pandemic [1] – to extremely severe, as in the case of the 1918 pandemic, which is estimated to have killed at least 20 million people worldwide [2-4]. Worryingly, the H5N1 avian influenza strain that is continuing to circulate in bird populations in South-East Asia [5] results in high mortality rates if contracted by humans, having an estimated case fatality ratio of 14-33% [6]. This strain could result in an extremely severe pandemic if it mutates or reassorts into a form transmissible between humans, a danger highlighted by research demonstrating the potential of H5N1 to mutate into a form readily transmissible between a mammal species, namely ferrets, a commonly used animal model for influenza transmission [7-9]. The planning of mitigation measures capable of reducing the number of infections and deaths resulting from a future influenza pandemic is therefore highly important.

A key strategy to mitigate a future pandemic would involve social distancing and antiviral measures and their continuation until a vaccination campaign has produced a cohort of immune individuals sufficient to prevent transmission [10-12]. Social distancing and the use of antiviral agents have the property that they can be activated rapidly, but do not confer any lasting immunity. Vaccination, on the other hand, does produce immunity, but a matched vaccine produced specifically for a newly emerged influenza strain will not be immediately available, as demonstrated in 2009.

The effectiveness and cost effectiveness of this plausible strategy depends upon several key factors. Firstly, the severity of a pandemic, as characterised by the proportion of symptomatic cases resulting in hospitalisation or death, not only directly determines the number of severe illnesses and deaths resulting from the pandemic, but also contributes to the total cost of the pandemic through medical costs and future productivity losses due to death. A second key factor is the duration for which social distancing and antiviral interventions are sustained. Interventions of longer duration have the capacity for greater reductions in attack rate and consequent deaths, however previous studies have shown school closure and workforce reduction intervention measures may result in large costs stemming from productivity losses [13-16]. A third key factor is the timing of the arrival of the pandemic relative to the start of a vaccination campaign. The experience of the 2009 pandemic indicated a period of six months between the identification of the pandemic virus strain in April and the first availability of a vaccine in October. The time taken for the virus to spread from North America, where it was first identified, to different parts of the world varied from 0 to 6 months, showing that most of the global population was potentially exposed to the virus before a suitable vaccine became available.

No previous studies explicitly address the realistic combination of rapidly activated social distancing and antiviral interventions together with vaccination, which is needed to deal with the probable 6-month delay in vaccine availability. This modelling study directly addresses this scenario, and seeks to quantify the cost effectiveness of a plausible range of combined social distancing, antiviral and vaccination intervention strategies. Furthermore, this study determines the effect which pandemic severity has on the cost effectiveness of intervention strategies. Pandemic severity is a key factor in the assessment of alternative intervention strategies, as strategies that are considered too costly and socially disruptive for mild pandemics similar to the 2009 pandemic may be optimal for severe pandemics with high mortality rates.

## Methods

A detailed, individual-based simulation model of a community in Western Australia (Albany, population ~30,000) was constructed to simulate the dynamics of an influenza pandemic. Comparing simulations with and without interventions allow the determination of its effect on the health of each individual and hence the overall attack rate. Data produced by the model determines health outcomes of each individual in the model, involving hospitalisation, ICU treatment, and death. Together with productivity losses, these outcomes were used to estimate intervention cost and cost effectiveness.

The model was developed using census, state, and local government data to construct contact networks involving households, schools and workplaces. Each household in the modelled community uniquely identified individuals in age groups (0–5, 6–12, 13–17, 18–24, 25–44, 45–64, 65+). Children were allocated to schools and classes, and adults to workplaces. Potentially infections contacts were modelled as occurring in households, school classes, workplaces, and randomly in the community. Three basic reproduction numbers (R_0_ = 1.5, 1.9, 2.7) and three case fatality rates (CFR = 0.03%, 0.1%, 1.5%) were used to capture pandemics with mild, moderate, and extreme characteristics. A full description of the model appears in an additional file [see Additional file 1, model parameter values are summarised in Additional file 1: Table S1.1 of that file].

### Three pandemic scenarios

Three plausible pandemic scenarios (mild, moderate and extreme pandemics) were defined in this study based on the transmissibility characteristics and severity of past pandemics. The transmissibility of a pandemic is defined in terms of its basic reproduction number R_0_ and associated illness attack rate. The severity of a pandemic is defined in terms of the case fatality rate.

Each simulation is assumed to begin when the first and subsequent cases arrive in the local community. After this point in time, one randomly located infected individual is seeded into the population each day, for the duration of the epidemic. Analysis shows that this rapidly triggers a local epidemic; within two weeks in the low-transmissibility scenario, and within one week for the higher transmissibility scenarios.

The CDC pandemic severity index uses the case fatality rate (CFR) for categorising pandemic severity [17]. This index was designed to better forecast the impact of a pandemic and was intended to allow recommendations on the use of intervention strategies to match the severity of future influenza pandemics. Severity categories were proposed from category 1, with a CFR < 0.1% to category 5, with a CFR >= 2.0%.

In this study, a mild pandemic was defined as having a reproduction number R_0_ of 1.5 and a CFR of 0.03%. These parameters were chosen as similar to the H1N1 2009 pandemic, which had an estimated reproduction number between 1.2 and 1.5 [18-20] with a CFR between approximately 0.01% and 0.08% [1]. A moderate pandemic was defined as having a reproduction number R_0_ of 1.9 and a CFR of 0.25%. The reproduction number of the 1957 and 1968 pandemics has been estimated to be in the range 1.5 and 2.0 [21-23] with CFRs between 0.03% and 0.16% [2,3]. An extreme pandemic was defined as having a reproduction number R_0_ of 2.7 and a CFR of 1.5%, to reflect the estimated transmissibility and severity characteristics of the 1918 pandemic, thought to have had reproduction number between 2.0 and 2.9 [22,24-26] with an estimated CFR between 0.74% and 1.8% [2-4].

### Vaccination

Simulation experiments were conducted to quantify the effect of vaccination combined with social distancing and antiviral treatment and prophylaxis interventions. In this study, a single-dose vaccination strategy was considered for mild pandemics. Clinical trials of new vaccines have shown that a single dose vaccine can induce significant immunity against the H1N1 2009 influenza strain [27]. A two-dose vaccination strategy is considered for moderate and extreme pandemics, assuming that individuals are naïve to future ‘serious’ influenza strains and that a two-dose vaccine is essential to achieve immunity [28-30].

For the H1N1 2009 pandemic, the first supplies of vaccines become available after five or six months following the appearance of the new strain of H1N1 influenza. In this study, a 6 months delay from the onset of the pandemic to the initiation of a vaccine campaign is assumed, as is a vaccination rate of 1% of the population per day. Sensitivity analyses of these assumptions were also conducted.

In this study, use of a highly effective vaccine is assumed. Trials of candidate vaccines for the H1N1 2009 pandemic influenza showed seroconversion rates of vaccines (defined as having a fourfold neutralizing seroconversion rate) between 82 and 92 per cent [27]. Vaccines with an efficacy of 75% are therefore assumed. A sensitivity analysis of this assumption is also conducted assuming vaccine efficacy of 65% and 85%. It is also assumed that the vaccination campaign will continue until the local epidemic effectively ceases, by creating a cohort of vaccine immune individuals. An assumption of full vaccination coverage is made, and a detailed sensitivity analysis of this assumption with vaccination coverage levels of 10% to 100% of the population also presented. Further details of the vaccine model can be found in an additional file [see Additional file 1.

It was assumed that vaccination is prioritised so that age groups known to have higher transmission rates would be vaccinated first. Previous modelling results have indicated that a transmitters-first vaccination strategy is more effective in reducing both attack and mortality rates than a vulnerable-first approach [31,32].

### Social distancing and antiviral drug interventions

A range of social distancing and antiviral intervention strategies including school closure, antiviral drugs for treatment and prophylaxis, workplace non-attendance (workforce reduction), and community contact reduction have been examined. Antiviral drug and social distancing interventions were initiated when specific threshold numbers of symptomatic individuals were diagnosed in the community, and this triggered health authorities to activate the intervention response. This threshold was taken to be 0.1% of the population. It was assumed that 50% of all symptomatic individuals were diagnosed, and that this diagnosis occurred at the time symptoms appeared. This intervention activation threshold occurs 13, 9 and 7 days after the simulations start in the mild, moderate and extreme pandemics respectively.

Antiviral and social distancing interventions are considered in combination with vaccination, and social distancing interventions are considered for either sustained periods (that is, until the local epidemic effectively ceases following creation of a cohort of immune individuals through vaccination) or periods of fixed duration (2 weeks or 8 weeks). For sustained school closure, all schools were closed simultaneously once the intervention trigger threshold was reached. For school closure durations of 2 weeks, which was only used for the mild pandemic scenario, and 8 weeks, which was used for all pandemic scenarios, schools were closed individually as follows: for a primary school the whole school was closed if 1 or more cases were detected in the school; in a high school only the class members of the affected class were isolated (sent home and isolated at home) if no more than 2 cases were diagnosed in a single class; however if there were more than 2 cases diagnosed in the entire high school the school was closed. Note that these school closure policies were only activated after the community-wide diagnosed case threshold was reached; cases occurring in schools before this time did not result in school closure. This policy of triggering school closure based on epidemic progression avoids premature school closure which can reduce the effectiveness of limited duration school closure [33].

In this study, assumptions made when modelling interventions are given in Tables S1.1 which, together with a full description of the simulated interventions, is given in an additional file [see Additional file 1].

In this study, antiviral drugs used for treatment and prophylaxis of household members (of a symptomatic case) are combined with vaccination and social distancing interventions. It was assumed that 50% of symptomatic individuals would be identified for antiviral treatment and/or prophylaxis, and that treatment and prophylaxis would occur 24 hours after the appearance of symptoms.

School closure (SC) was modelled by assuming that when the intervention was in effect all school children stayed at home and did not make contact with class members, and that at least one supervising adult from each affected household also stayed at home. Workforce reduction (WR) was modelled by assuming that for each day the intervention was in effect each worker had a 50% probability of staying at home and thus did not make contact with co-workers. Community contact reduction (CCR) was modelled by assuming that on days when the intervention was in effect all individuals made 50% fewer random community contacts.

**Figure 1:**
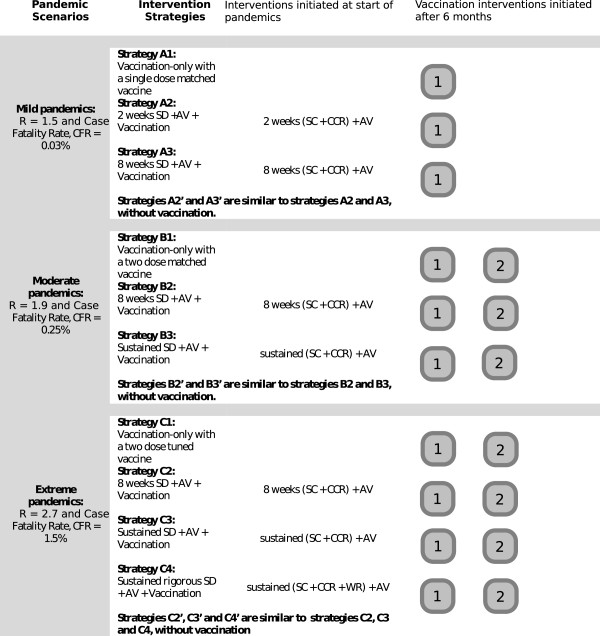
**Synopsis of pandemic scenarios and intervention strategies.** A synopsis of three pandemic scenarios and plausible intervention strategies. Social distancing (SD) intervention indicates school closure (SC) and community contact reduction (CCR). Rigorous social distancing indicates the addition of workforce reduction (WR) to the SC and CCR interventions.

A summarised description of the simulated interventions for three pandemic scenarios is presented in Figure 1. Previous modelling studies have shown that combinations of social distancing and antiviral interventions are capable of significantly reducing the final attack rate of an influenza pandemic [34-40]. A strategy of school closure and community contact reduction combined with antiviral treatment and household prophylaxis was therefore chosen as a plausible representative of these intervention strategies. Different durations of social distancing were chosen according to the severity of each pandemic scenario, on the grounds that the duration of disruptive social distancing measures tolerated (or demanded) by the public would depend upon the perceived severity of the pandemic. 8 weeks of social distancing was examined for all scenarios; for mild pandemics 2 weeks of social distancing was examined (which corresponds to a strategy employed during the 2009 pandemic in several countries), while for moderate and extreme pandemics sustained social distancing was also included. All social distancing strategies were examined with and without vaccination.

### Influenza transmission model

The number of contacts made by each individual each day in school, work and community settings were adjusted to reproduce the proportion of cases occurring in different settings as reported by empirical studies, specifically 40% of infections occurred in households, 30% in schools and workplaces, and 30% in the wider community [34,41,42]. Contacts within schools and workplaces occurred in fixed-size mixing groups of maximum size 10; within mixing groups contact was assumed to be homogeneous. Community contacts occurred between randomly selected individuals, weighted toward pairs of individuals with nearby households. The mixing group sizes, and location-specific distribution of where infection occurs, are given in an additional file [see Additional file 1, in Tables S1.1 and S1.2 respectively.

Following each contact a new infection state for the susceptible individual (either to remain susceptible or to become infected) was randomly chosen via a Bernoulli trail [43]. Once infected an individual progressed through a series of infection states according to a fixed timeline.

The probability that a susceptible individual would be infected by an infectious individual was calculated according to the following transmission function, which takes into account the disease infectivity of the infectious individual *I*_*i*_ and the susceptibility of susceptible individual *I*_*s*_ at the time of contact.

$$\begin{array}{l} P_{\mathrm{trans}}\left(I_{i},I_{s}\right)=\beta \times \mathrm{Inf}\left(I_{i}\right)\times \mathrm{Susc}\left(I_{s}\right)\times \mathrm{AVF}\left(I_{i},I_{s}\right)\\ \;\times \mathrm{Vaccine}\left(I_{s}\right) \end{array}$$

Each factor contributing to the transmission probability (basic transmissibility *β*, time-varying transmissibility *Inf*(*I*_*i*_), age-based susceptibility *Susc*(*I*_*s*_), antiviral effectiveness *AVF*(*I*_*i*_, *I*_*s*_), and vaccine effectiveness *Vaccine*(*I*_*s*_)) is described in detail in an addition file [see Additional file 1. The transmission probability coefficient β, capturing the infectivity of the virus strain, was chosen to give unmitigated epidemics with a specific effective reproduction number R, and R = 1.5, 1.9 and 2.6 have been used in this study to capture transmission characteristics for mild, moderate and extreme influenza pandemics respectively. The R_0_ values for the three pandemic scenarios were calculated by fitting an exponential growth curve to the daily incidence in the early stages of the pandemic, using the daily incidence and serial interval distribution recorded from 40 randomly seeded simulations, following the method described in [44].

Age-based susceptibility *Susc*(*I*_*s*_) was calibrated to reproduce age-specific infection rates observed in the 2009 pandemic [45]. Additional file 1: Figure S1.1 appearing in an addition file [see Additional file 1 gives the age-specific distribution of infections for the three pandemic scenarios. An alternative age-based susceptibility profile, where all age groups were equally susceptible, was used in a sensitivity analysis.

Epidemics were initiated by introducing one randomly located infected seed individual into the population each day, for the duration of the epidemic. All simulations were repeated 40 times with random numbers controlling the outcome of stochastic events (the locality of seeded infected individuals and the probability of transmission) and the results were averaged. Analysis of this simulation model has shown that the 40-run mean attack rate is highly unlikely (95% confidence) to differ by more than 1.2% of the mean attack rate of a much larger set of experiment repeats.

### Health outcomes

Calculation of costs arising from lost productivity due to death and from hospitalisation of ill individuals requires that individual health outcomes (symptomatic illness, hospitalisation, ICU admission, and death) be estimated for each pandemic scenario and for each simulated strategy level. H1N1 2009 pandemic data from Western Australia was used to provide this relationship between the mortality rate and numbers requiring hospitalisation and ICU care. These data indicated a non-ICU hospitalisation to fatality ratio of 32:1 and an ICU admission to fatality ratio of 3:1. These values align with those in a previous study for the H1N1 2009 pandemic by Presanis et al. in [46] which estimated the ratios in the ranges 17–37 to 1 and 3.1–5.0 to 1, respectively.

### Economic analysis

The economic model translates the age-specific infection profile of each individual in the modelled population, as derived by the Albany simulation model, into the overall pandemic cost burden. This overall cost comprises the following components: costs arising directly from interventions including social distancing costs, antiviral costs and vaccination costs (this vaccination cost includes the cost of a vaccine itself, delivery cost of vaccines, and time and travel cost required to obtain vaccines); loss of productivity in the workplace arising from illness; costs associated with hospitalisation of ill individuals; and productivity losses due to death.

In this study, the approach taken determines the total economic cost to society incurred during a future influenza pandemic. Total costs involve both direct healthcare costs (e.g. the cost of medical attention due to a GP visit, or for hospitalisation) and costs due to productivity loss. Pharmaceutical costs (i.e. costs related to antiviral drugs and vaccines) are also estimated. All costs are reported in 2010 US dollars using consumer price index adjustments. 2010 US dollar values are used to make the results readily convertible to a wide range of developed countries. A full description of the economic analysis, including cost data used in establishing the overall cost of pandemic scenarios is given in an additional file [see Additional file 1].

### Cost effectiveness

The cost effectiveness of a given intervention strategy is presented in terms of cost per Life Years Saved (LYS). The numerator used in this cost effectiveness ratio was derived from the total cost arising from a given intervention being applied to the whole community. The denominator was calculated as the difference between years of life lost for an unmitigated pandemic and a pandemic with the intervention applied. Years of life lost data were derived for each simulation from the ages and life expectancies of the (age-specific) individuals who died as a result of the pandemic.

The cost effectiveness of each intervention is presented as a cost in dollars ($) per LYS per person. This was derived by establishing the total cost for a particular intervention strategy and then dividing it by the population of the Albany model, approximately 30,000 individuals, so allowing the results to be applied to a population of any size.

With influenza, where individuals suffer a reduced quality of life for a short period (in comparison with lifespan), this Life Year Saved measure is approximately equivalent to the commonly used Quality Adjusted Life Years (QALY) gained measure.

## Results

**Figure 2:**
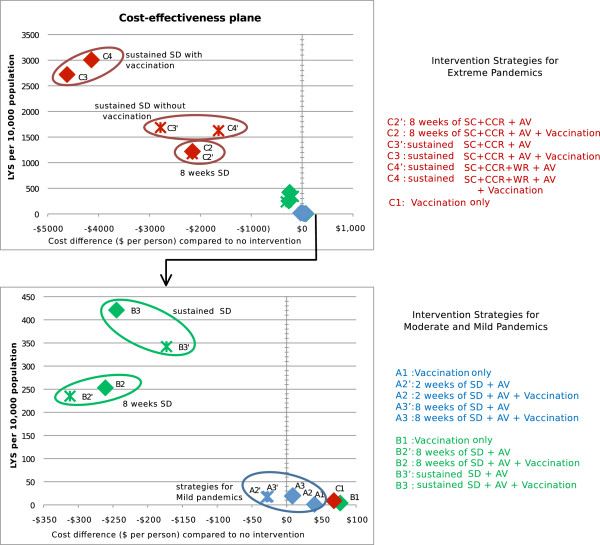
**Cost effectiveness plane for intervention strategies.** Each intervention strategy is plotted with horizontal position according to relative cost in dollars per member of population compared to no intervention, and with vertical position according to the number of life-years saved (LYS) per 10,000 population. The lower figure contains an enlarged view around the axis to clarify the position of interventions for moderate and mild pandemics. Colours denote pandemic severity: red for extreme (R_0_ = 2.7, CFR = 1.5%), green for moderate (R_0_ = 1.9, CFR = 0.25%) and blue for mild (R_0_ = 1.5, CFR = 0.03%). Crosses indicate interventions without vaccination, diamonds indicate vaccination interventions. Interventions are labelled as for Figure 1, abbreviations used in the figure legend are SC for school closure, CCR for community contact reduction, AV for antiviral treatment and household prophylaxis, and WR for workforce reduction.

The effectiveness in terms of attack rate reduction and life-years saved, the total cost, and the cost per life-year saved ratio of each intervention strategy is shown in Table 1. Figure 2 shows a cost effectiveness plane which plots each intervention horizontally according to cost (compared to no intervention) and vertically according to effectiveness (life years saved). Incremental cost effectiveness ratios are given in an additional file [see Additional file 2].

**Table 1:** Effectiveness, cost and cost effectiveness of interventions for mild, moderate and extreme pandemics

**Mitigation strategies**	**Attack rate (%)**	**Life years saved per 10000**	**Total cost ($) per person**	**Cost ($) per LYS**
**Mild Pandemics (R=1.5 and CFR=0.03%)**				
No intervention	14	-	$170	$0
Strategy A1: Vaccination-only	13.5	1*	$210	-
Strategy A2^′^: 2 weeks of SD + AV	5	17	$141	$83686
Strategy A2: 2 weeks of SD + AV + Vaccination	4	19	$178	$93202
Strategy A3^′^: 8 weeks of SD + AV	5	18	$143	$81500
Strategy A3: 8 weeks of SD + AV + Vaccination	3	20	$179	$89574
**Moderate Pandemics (R=1.9 and CFR=0.25%)**				
No intervention	33	-	$1,031	$0
Strategy B1: Vaccination-only	32.5	4*	$1,108	-
Strategy B2^′^: 8 weeks of SD + AV	16	235	$719	$30527
Strategy B2: 8 weeks of SD + AV + Vaccination	15	253	$770	$30417
Strategy B3^′^: Sustained SD + AV	9	342	$858	$25085
Strategy B3: Sustained SD + AV + Vaccination	4	421	$786	$18659
**Extreme Pandemics (R=2.7 and CFR=1.5%)**				
No intervention	44	-	$6,953	$0
Strategy C1: Vaccination-only	43.8	9*	$7,021	-
Strategy C2^′^: 8 weeks of SC+CCR + AV	29	1176	$4,794	$40757
Strategy C2: 8 weeks of SC+CCR + AV + Vaccination	28	1219	$4,797	$39340
Strategy C3^′^: Sustained SC+CCR + AV	22	1686	$4,169	$24731
Strategy C3: Sustained SC+CCR + AV +Vaccination	11	2720	$2,332	$8571
Strategy C4^′^: Sustained SC+CCR+WR + AV	22	1625	$5,310	$32671
Strategy C4: Sustained SC+CCR+WR + AV +Vaccination	7	3006	$2,812	$9355

For each pandemic severity and intervention strategy Table 1 gives the symptomatic attack rate as a percentage of the population, the number of life years saved (LYS) compared to no intervention (expressed as LYS per 10,000 population), the total cost of the pandemic per person in the community, and the cost per LYS per person. Interventions are abbreviated as follows: SD – social distancing (a combination of school closure and community contact reduction), AV – antiviral treatment and household prophylaxis, SC – school closure, CCR – community contact reduction, WR – workforce reduction.

* indicates LYS value not statistically significantly different from zero due to stochastic simulation variation – cost per LYS values with insignificant denominator have been omitted.

**Figure 3:**
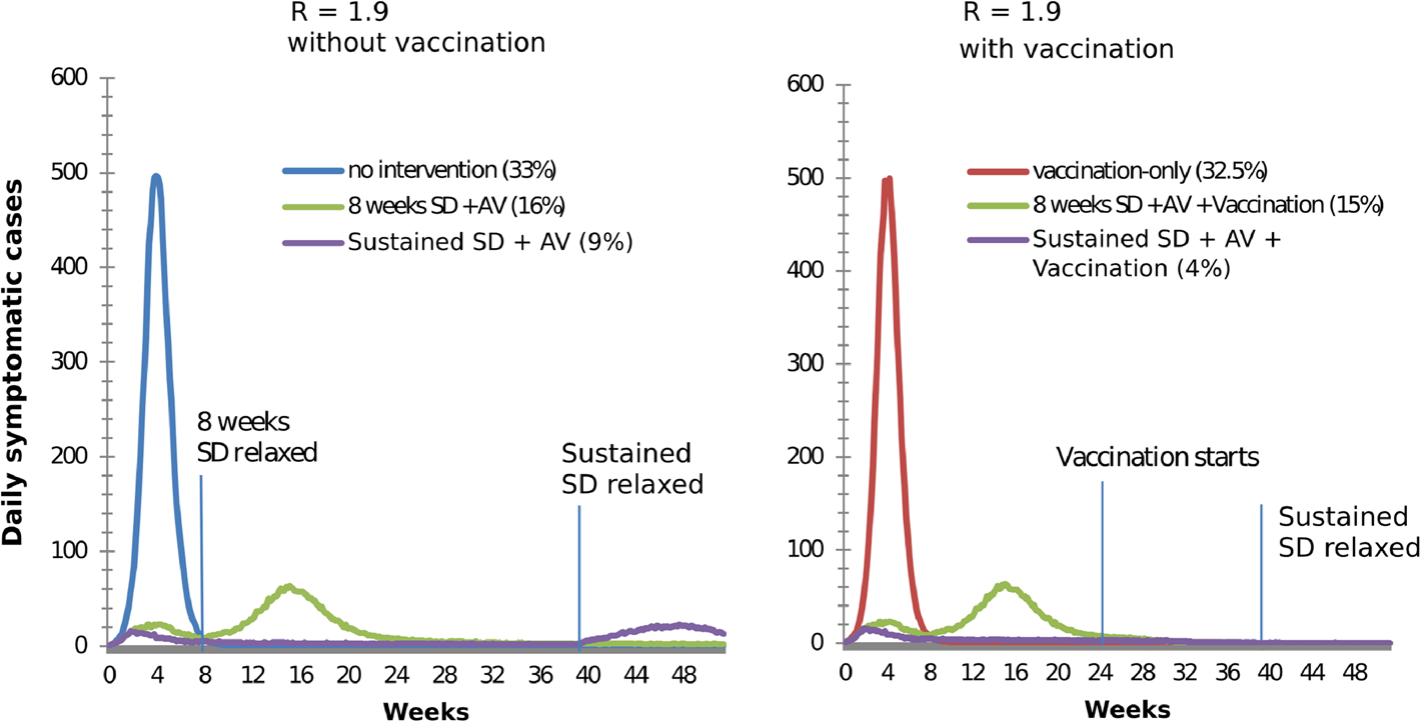
**Epidemic curves with and without vaccination for moderate pandemics.** Daily incidence curves are shown for six intervention strategies for a mild pandemic (R_0_ = 1.9) Figure legend text. The left panel shows three strategies that do not include vaccination while the right panel shows three strategies than include vaccination. The composition of each strategy is given in the figure legend along with the final attack rate for that strategy. Points along the horizontal (time) axis where social distancing interventions are relaxed or vaccination begins are marked with a vertical line.

The results suggest that for moderate and extreme pandemics with CFR of 0.25% and 1.5% respectively, a strategy of sustained social distancing and antiviral treatment and prophylaxis is highly cost effective, reducing both mortality and total costs. Furthermore, the addition of vaccination to sustained social distancing further reduces both mortality and total costs. For moderate pandemics vaccination is a cost effective compliment to long duration social distancing, but not social distancing of limited duration. For mild pandemics, limited duration social distancing was found to be cost effective, but vaccination was not cost effective either as a sole intervention or as an accompaniment to limited duration social distancing,

The use of vaccination as a sole intervention resulted in total costs that are higher than for the no intervention scenario without a compensating reduction in mortality. Vaccination was therefore not cost effective as a sole intervention strategy for any pandemic scenario when measured in terms of cost per life years saved. Figure 3, which shows daily incidence curves for interventions with and without vaccination illustrates why this is the case: the unconstrained growth, peak and subsidence of the pandemic occur well before the vaccination campaign begins.

As with all modelling studies, the numerical results presented here depend upon assumptions and model parameters which are not known exactly. However, the results presented below highlight the relative cost effectiveness of alternative intervention strategies that differ only on intervention parameters and for which all the other model parameters are the same. These results should be robust to plausible variations in model parameter values, as such variations in model parameter values will influence both simulation outcomes in the same way. In addition, sensitivity analyses for the parameter values most likely to have a major impact on the outcome were conducted, the results of these are presented at the end of the results section.

### Extreme pandemics (R_0_ 2.7, CFR 1.5%)

**Figure 4:**
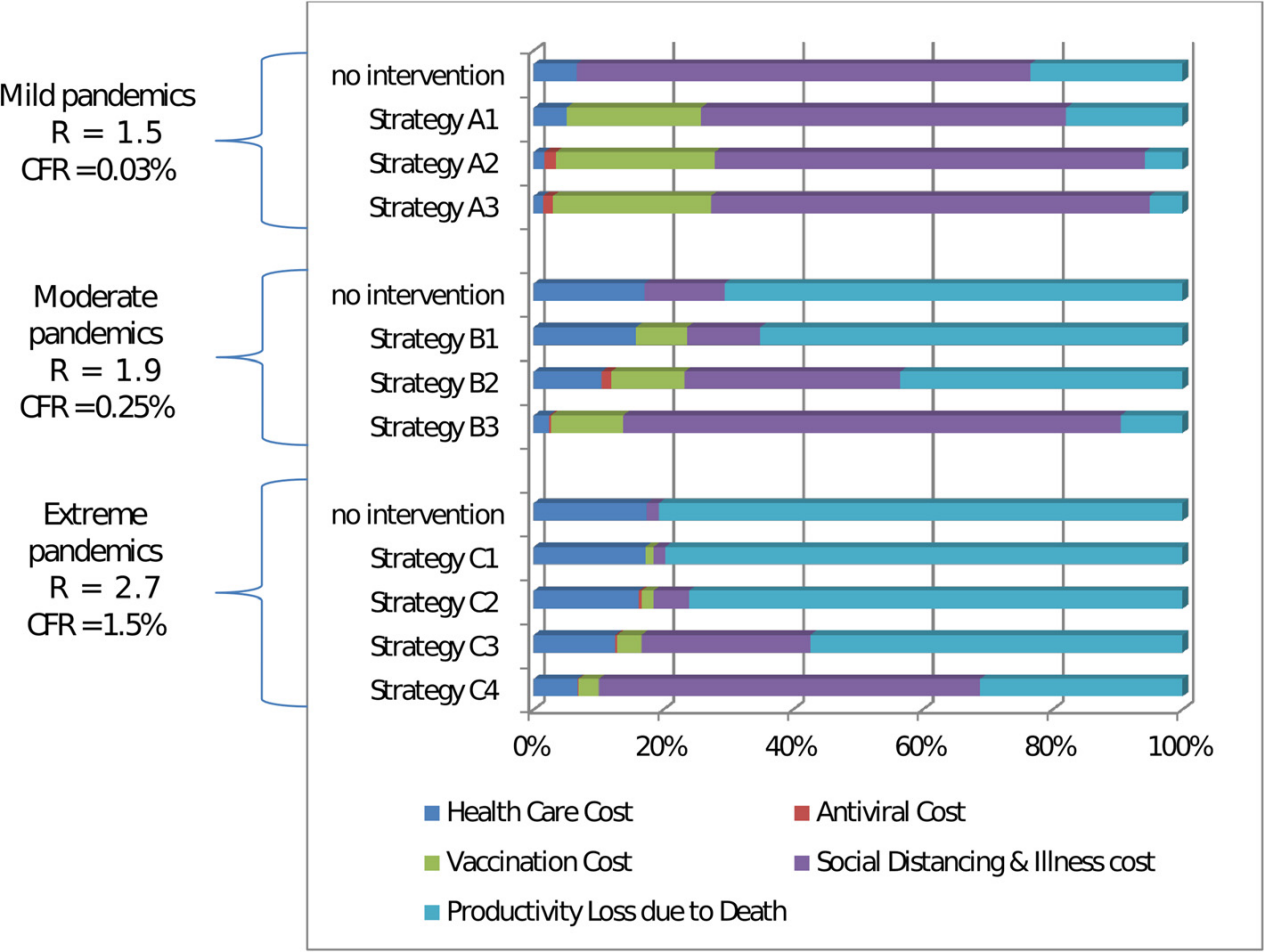
**Cost component breakdown for intervention strategies.** The total cost for each intervention strategy and pandemic severity scenario is broken down into 5 component costs, with the width of each coloured bar indicating the percentage of the total cost constituted by each component – health care costs (dark blue), including GP visits and hospitalisation; antiviral costs (red) including pharmaceutical, dispensing and stockpile renewal costs; vaccination costs (green) including vaccine development, production and distribution; lost productivity due to social distancing and illness (purple); and productivity loss due to death (light blue). costs and the percentage of each component is indicated by a coloured bar. Note that all total costs are scale to have the same width, so absolute widths are not comparable between strategies.

For extreme pandemics, the results indicate that the most effective and cost effective strategies included vaccination combined with sustained school closure and community contact reduction plus antiviral treatment and household prophylaxis. These strategies (labelled C3 and C4 in Figure 2 and Table 1), were highly effective due to the fact that the combination of rigorous social distancing and antiviral medication is capable of suppressing infection transmission until vaccination creates a sufficient cohort of immune individuals, effectively halting the pandemic. As a result these strategies reduced the number of symptomatic infections by three quarters compared to an unmitigated pandemic, and the consequent reduction in mortality resulted in a large number of life years saved. A further consequence of this reduction in mortality was that these highly effective interventions also had the lowest total cost, due to the fact that costs, for extreme pandemics, are dominated by death-related productivity losses. This can be seen in Figure 4, which shows the breakdown of cost components for the different pandemic and intervention scenarios. Strategy C4, which adds sustained social distancing to strategy C3, saved approximately 10% more life years compared to C3, and cost 17% more.

The next most effective strategies were those that combined sustained school closure and community contact reduction plus antiviral treatment and household prophylaxis, but which did not include vaccination (C3^′^ and C4^′^ in Figure 2 and Table 1). The omission of vaccination results in a resurgence of the pandemic once social distancing interventions are relaxed, which can be seen in Figure 3. Consequently, these strategies could reduce the attack rate by at most 50%, resulting in fewer lives saved and higher costs. The omission of vaccination thus rendered long duration social distancing less cost effective.

Strategies that include limited duration social distancing (C2 and C2^′^, which include 8 weeks social distancing) were less effective and less cost effective than strategies with sustained social distancing, irrespective of whether vaccination was included. These strategies saved fewer life years than those that include sustained social distancing, and had a higher cost in terms of cost per LYS. 8 weeks social distancing temporarily slowed the pandemic spread while in effect, however the pandemic resumed once intervention measures were relaxed, and the final attack rate was reduced by at most 36%. This is illustrated in Figure 3, which shows that the initial growth of the pandemic, and its resurgence once social distancing interventions were relaxed, both occur before vaccination was initiated.

### Moderate pandemics (R_0_ 1.9, CFR 0.25%)

For moderate pandemics, the most effective strategies were sustained social distancing and antiviral measures; these are strategies B3 and B3^′^ which appear at the top of Figure 2 (lower panel). These strategies saved a significant number of lives, and reduced the total cost of the pandemic, making them highly cost effective. As for the case of extreme pandemics, the addition of vaccination to sustained social distancing (strategy B3) prevents a resurgence of the pandemic once social distancing measures are relaxed, reducing the final attack rate by a factor of 2. This resulted in an additional saving of approximately 20% more life years, and further reduced the total cost, making vaccination a cost effective accompaniment to sustained social distancing for moderate pandemics.

Strategies based upon limited duration (8 weeks) social distancing and antiviral measures were less effective but were also less costly than those with sustained social distancing; these are strategies B2 and B2^′^ in Figure 2 and Table 1. The addition of vaccination to limited duration social distancing (strategy B3) was not highly cost effective, costing approximately $50 more per person but saving only 7% more life years.

### Mild pandemics (R_0_ 1.5, CFR 0.03%)

Due to the low case fatality rate (CFR) for mild pandemics, all interventions resulted in a small number of life years saved (LYS). Because of the small number of LYS, cost per LYS is a less informative measure of cost effectiveness for mild pandemics. The cost effectiveness of interventions for mild pandemics are therefore described below in terms of total pandemic costs and attack rate reductions, rather than cost per LYS.

For a mild pandemic, a strategy of 2 or 8 weeks social distancing (school closure plus community contact reduction) combined with antiviral treatment and household prophylaxis reduced the AR by more than half. The direct cost of these intervention strategies is offset by reduced productivity losses due to reduced illness, resulting in a small net reduction in total pandemic cost.

Adding vaccination to the 2 or 8 weeks social distancing and antiviral strategies resulted in an additional 1% or 2% reduction in attack rate respectively, at an additional cost of approximately $40 per person. A vaccination-only strategy increased the total pandemic cost by $40 per person, but reduced the attack rate by only 0.5%.

### Sensitivity analysis

Sensitivity analyses were conducted to assess key parameters related to the vaccination strategies and how the results depend upon the parameters settings. Alternative parameter values for vaccination delay, vaccination rate, vaccination coverage, vaccine efficacy and age-specific susceptibility were examined. Significant findings are summarised below; full details appear in an addition file [see Additional file 2].

Alternative delays to the activation of vaccination of 2, 4 and 6 months were analysed in conjunction with social distancing durations of 2, 4 and 6 months (all 9 combinations were simulated for moderate and extreme pandemics). It was found that vaccination was much more effective and cost effective if social distancing interventions continued until at least the start of the vaccination campaign. For example, strategy B3 (2 months social distancing and antiviral measures plus vaccination starting after 6 months) resulted in a final attack rate of 14.9%, whereas if vaccination started after only two months, the final attack rate was 6.3%.

Increasing the vaccination rate was beneficial, reducing both final attack rate and total cost when social distancing was relaxed at the beginning of the vaccination campaign, particularly for pandemics with high transmissibility.

Sensitivity analyses show that decreasing the vaccination delay from 6 months to 2 months and / or accelerating vaccination rate from 1% to 5% per day did not render vaccination-only intervention strategies cost-effective. Smaller vaccination delays and increased vaccination rates only marginally reduced the attack rate and total cost.

In the main results it was assumed that individual susceptibility to infection differed by age, resulting in age-specific infection rates similar to the 2009 pandemic, where 18–24 years olds had the highest attack rates while those 25 years and older had the lowest [45]. Previous pandemics have exhibited different age-specific attack rate profiles. The 1957 pandemic resembled seasonal influenza with the highest attack rates in children, while the 1968 pandemic had similar attack rates in all age groups [3]. The sensitivity of the results to an alternative assumption that all age groups would be equally susceptible was examined. The result was a shift in the burden of illness to older age groups, and as a result, slightly fewer (less than 12%) life years were saved by interventions. However the shift of illness to older age groups also reduced the death-related productivity losses, resulting in lower total pandemic costs and slightly improved cost effectiveness of interventions.

## Discussion

From a public health perspective a combination of antiviral treatment and household prophylaxis with sustained social distancing is effective and cost effective for moderate and extreme pandemics, reducing both mortality and total cost. This is because at high severity measures that save lives also reduce large death-related productivity losses. The addition of vaccination at 6 months post pandemic initiation further reduces the attack rate, as vaccination eliminates the resurgence of the pandemic that would otherwise occur when social distancing measures are relaxed. Consequentially, mortality and the overall cost are also reduced, making strategies that couple vaccination with sustained social distancing the most effective and cost effective strategies for moderate and severe pandemics.

Importantly, it is shown that in order to obtain significant life-saving benefits from vaccination, public health social distancing interventions must be (a) rigorous enough to significantly suppress transmission, and (b) continued until they overlap in time with the vaccination program, to the extent that there is a sufficient cohort of immune individuals capable of effectively halting pandemic transmission (i.e. reducing the effective reproduction number to less than 1). While these results do not quantify the optimal duration of social distancing, the sensitivity analyses performed indicate that the point at which social distancing interventions can be safety dropped without risking a pandemic resurgence depends upon pandemic severity and transmissibility, as well as vaccination delay, vaccination rate, and vaccination efficacy.

For mild pandemics the addition of vaccination to any intervention strategy always increases the total cost, yet saves few additional lives. As a consequence, for pandemics known to be mild, the development and production of a vaccine for a mass vaccination program is not cost effective, having an incremental cost effectiveness ratio (ICER) in excess of $60,000 per LYS. For comparison, the UK National Institute for Health and Clinical Excellence (NICE) uses a range of between $31,000 and $48,000 (20,000 to 30,000 GBP) per Quality Adjusted Life Year saved (QALY) as a threshold below which an intervention can be deemed cost effective enough to warrant public subsidy via the National Health Service (NHS) [47]. For non-chronic conditions such as influenza, QALYs and LYS are effectively equivalent. Development of a vaccine may still be worthwhile, allowing it to be used for highly vulnerable groups, or for inclusion in a future seasonal influenza vaccine. For mild pandemics, antiviral measures coupled with social distancing interventions of limited duration can however be effective and cost effective.

The results suggest that if severity is unknown, which is likely at the beginning of a newly emerged pandemic, social distancing and antiviral measures should be adopted. If severity is found to be mild, social distancing measures may be dropped, having reduced the illness attack rate without incurring a large net cost. If however the pandemic is found to be severe, production and delivery of vaccines is of primary importance, and the timing of this should determine for how long rigorous social distancing should continue.

It should be noted that for the intervention strategies considered in this study, sustained social distancing is assumed to continue until the vaccination campaign is complete (or for an equivalent duration for strategies without vaccination). Social distancing of this duration (greater than 6 months) is unprecedented and may be practically impossible. However, the results for the strategies that include sustained social distancing are important because they highlight a significant distinction between moderate and extreme pandemics. In contrast to mild and moderate pandemics, for extreme pandemics the results demonstrate that even if social distancing cannot be sustained indefinitely, longer periods of social distancing are strictly better than shorter periods, resulting in fewer lives lost and a lower total cost, with or without vaccination. This can be seen by comparing strategies C1, C2 and C3 (also no intervention, C2^′^ and C3^′^) in Table 1. This indicates that for extreme pandemics, public health efforts to sustain social distancing for as long as possible are worthwhile for both humanitarian and economic reasons, if a long-term, whole-of-society perspective is taken.

For some pandemic scenarios the selection of strategy represent a cost effectiveness trade-off. In these cases the choice of intervention strategy allowed greater effectiveness to be purchased for a greater cost, which can result in both strategies having a similar cost effectiveness ratio. This can be seen, for example, in the choice to add sustained workforce reduction to sustained social distancing and antiviral measures together with vaccination for extreme pandemics. It is important to note that where such a cost effectiveness trade-off is being considered, two interventions can have similar cost effectiveness as judged by the cost per LYS ratio while differing on the number of life years saved. In such cases where cost effectiveness ratios are in a similar range, the reduction in attack rate (i.e. effectiveness of interventions) and consequent reduction in mortality will be the key factor to be used by public health authorities when choosing between two such strategies.

The contact structure and behavioural assumptions of our simulation model are based on an Australian context. However, comparing the results of studies that use our simulation model [31,33,39,40] to a variety of other individual-based simulation models at a variety of scales (e.g. small community [37], city [38], country [34,48,49]) shows that the results of this Australian community model are consistent with these other models, in as far as comparable pandemic and intervention scenarios are being evaluated. We thus believe that the model is broadly representative of developed world cities, and the results are thus applicable to US, European or other developed world populations.

### Related research

The studies of Andradottir et al. [50], Prosser et al. [51], and Khazeni et al. [52] examined the cost effectiveness of vaccination for a pandemic with the severity and transmissibility characteristics of the 2009 pandemic, which corresponds to the mild scenario in this study. Each of these studies found that vaccination could be cost effective if initiated at the beginning of the pandemic, but that delays in vaccination greatly reduced effectiveness and cost effectiveness. The study of Andradottir et al. for example found that with a 2 month delay, vaccination had no meaningful effect, which is consistent with the current study’s findings. The studies of de Blasio et al. [53] and Conway et al. [54], which examined the effectiveness of vaccination for a 2009-like pandemic, also found the effectiveness of vaccination to fall rapidly with increasing vaccination delay, although they did not analyse costs.

The study of Newall et al. [55] considered a range of pharmaceutical (vaccination and antiviral) intervention strategies for the scenario of a severe pandemic, with CFR = 1% in adults. The study included vaccination with a matched vaccine that was delayed 6 months from the start of the pandemic. It was found that this strategy was effective in reducing the final attack rate and mortality if accompanied by a pre-pandemic vaccination campaign that started at the beginning of the pandemic; but that the effectiveness of this combined vaccination strategy degraded if the pre-pandemic vaccination campaign was delayed, highlighting the need for some form of intervention at the beginning of a pandemic. Note that this study did not consider social distancing interventions.

## Conclusions

This study has quantified the effectiveness and cost effectiveness consequences of plausible public health pandemic influenza mitigation strategies, taking into account both the severity of the pandemic and the time-critical interplay between pandemic dynamics and intervention timing.

The use of vaccination to effectively mitigate pandemic influenza is shown to be complementary to interventions which can be activated more rapidly. Social distancing and antiviral interventions can be applied immediately, and can suppress transmission while in effect, whereas vaccination, once available in sufficient quantities, can create a cohort of immune individuals to effect control of the pandemic. Without social distancing and antiviral interventions, vaccination comes too late to make a difference; without vaccination, the inevitable relaxation of social distancing interventions results in a resurgence of the pandemic.

## Competing interests

GJM has received a travel grant from GlaxoSmithKline to attend an expert meeting in Boston, USA. JKK and NH have no potential competing interests.

## Authors’ contributions

GM, JK and NH were responsible for the conception and design of the simulation experiments. NH and JK were responsible for simulation and economic model implementation. NH conducted simulation experiments. All authors were involved in the analysis of simulation results and writing the manuscript. All authors read and approved the final manuscript.

## Pre-publication history

The pre-publication history for this paper can be accessed here:

http://www.biomedcentral.com/1471-2334/13/81/prepub

## Supplementary Material

Additional file 1 **Extended methods description.** Vaccination Timing Additional file 1.docx, Microsoft Word .docx format. Contains model parameter table and additional textual description of simulation and economic model. Click here for file

Additional file 2 **Sensitivity analysis and additional results.** Vaccination Timing Additional file 2.docx, Microsoft World .docx format. Contains sensitivity analysis results in textual and tabular form, and incremental cost effectiveness ratio (ICER) results. Click here for file
