# Supplementary material for: Vaccination strategies for future influenza pandemics: a severity-based cost effectiveness analysis
Source: BMC Infect Dis. 2013 Feb 11;13:81. doi: 10.1186/1471-2334-13-81 (PMC3637125; doi:10.1186/1471-2334-13-81)
Supplement: Additional file 2 — Sensitivity analysis and additional results. Vaccination Timing Additional file 2.docx, Microsoft World .docx format. Contains sensitivity analysis results in textual and tabular form, and incremental cost effectiveness ratio (ICER) results. [file 1471-2334-13-81-S2.docx]

# The Cost Effectiveness of Pandemic Influenza Mitigation Strategies Involving Vaccination – Additional File 2

# Sensitivity Analyses

Sensitivity analyses were conducted to assess key parameters related to the vaccination strategies. Alternative parameter values for vaccination delay, vaccination rate, vaccination coverage and vaccine efficacy were examined and the effect which these parameter settings have on the resulting attack rate, cost and the relative cost effectiveness of interventions were determined. Results of these sensitivity analyses for three pandemic scenarios are presented in Tables S2.1 – S2.3 and in Figures S2.1.

## Vaccination Delay and Vaccination Rate

Tables S2.1, S2.2 and S2.3 give illness attack rates, total costs and cost per LYS for each of the strategies in the main paper, for vaccination delays of 2, 4 and 6 months and for vaccination rates of 1% and 5% per day. Results for additional limited duration social distancing strategies, where social distancing is continued for 2, 4 or 6 months, are also given for moderate and extreme pandemics.

For mild pandemics (see Table S2.1), a shorter vaccination delay leads to somewhat lower final attack rate, with a delay of 2 months giving attack rates on the order of 2 percentage points lower than vaccination at 6 months. Total pandemic costs are only slightly reduced by shorter vaccination delays or increased vaccination rate. With the low case fatality ratio of mild pandemics, very few additional lives are saved by small reductions in attack rate, and as a result reduced vaccination delay and/or increased vaccination rate does not render vaccination cost effective for mild pandemics.

For moderate and extreme pandemics (see Tables S2.2 and S2.3), where long-duration social distancing interventions might be employed, reductions in vaccination delay and increases in vaccination rate potentially have a much more significant impact on effectiveness and cost effectiveness. These scenarios can be divided in to three categories.

The first are the scenarios (shaded blue in Tables S2.2 and S2.3) where there is no social distancing, or social distancing ends prior to the commencement of vaccination. In these cases the effectiveness of interventions is essentially the same as that of the non-vaccination interventions alone – vaccination occurs to late to have a significant impact on the pandemic.

The second set of scenarios (shaded green) are those where limited duration social distancing is employed, but is sustained until at least the start of the vaccination campaign. For these scenarios, vaccination provides a substantial reduction in illness, mortality and total pandemic cost, rendering vaccination highly cost-effective.

The third set of scenarios (shaded purple) with sustained social distancing (that is, social distancing is assumed to continue until the vaccination campaign is complete). For these scenarios, vaccination is an effective addition to non-vaccination interventions, since it eliminates any resurgence of the pandemic when social distancing interventions are relaxed. For these scenarios reduced vaccination delay does not lead to substantially lower attack rate; however total pandemic costs are reduced, since the expensive social distancing interventions are relaxed sooner.

The effectiveness of vaccination was found to be relatively insensitive to increasing the rate of vaccination. The greatest effect was in the scenarios where SD stopped exactly as vaccination began. For example, if 2 months SD and 2 months vaccination delay is considered, accelerating vaccination rate from 1% to 5% can further reduce the attack rate approximately by 20%-30. This is plausible, since in this situation the vaccination is effectively in a race against the resurgent pandemic transmission to create immune individuals. In all other scenarios, increasing vaccination rate from 1% to 5% per day reduced the attack rate by less than 1 percentage point. Increased vaccination rate did however substantially lower total costs in the sustained social distancing strategies, as social distancing interventions were relaxed sooner.

**Table S2.1** : Vaccination delay and vaccination rate sensitivity analysis for mild pandemics.

|  | | | Vaccination Rate 1% per day | | Vaccination Rate 5% per day | |
| --- | --- | --- | --- | --- | --- | --- |
| **Pandemic Scenarios** | Mitigation Strategies | Delay in Vaccination | Illness Attack Rate (%) | Total Cost ($) | Illness Attack Rate (%) | Total Cost ($) |
| **Mild Pandemics:**  **R = 1.5 and**  **CFR = 0.03%** | Strategy A1: Vaccination-only | 6 months | 13.5 | $210 | 13.3 | $209 |
|  |  | 4 months | 13 | $208 | 12.8 | $207 |
|  |  | 2 months | 11.2 | $201 | 10.3 | $197 |
|  |  |  |  |  |  |  |
|  | Strategy A2:  2 weeks SD +AV+Vacc | 6 months | 3.8 | $178 | 3.6 | $177 |
|  |  | 4 months | 3.1 | $174 | 2.9 | $173 |
|  |  | 2 months | 2.1 | $168 | 1.8 | $167 |
|  |  |  |  |  |  |  |
|  | Strategy A3:  2 months SD +AV+Vacc | 6 months | 3.4 | $179 | 3.2 | $178 |
|  |  | 4 months | 2.6 | $175 | 2.4 | $174 |
|  |  | 2 months | 1.6 | $167 | 1.3 | $163 |

**Table S2.2**: Vaccination delay and vaccination rate sensitivity analysis for moderate pandemics.

|  | | | **Vaccination Rate 1% per day** | | | | **Vaccination Rate 5% per day** | | | |
| --- | --- | --- | --- | --- | --- | --- | --- | --- | --- | --- |
| **Pandemic Scenarios** | Mitigation Strategies | Delay in Vaccination | Illness Attack Rate (%) | LYS per 10,000 | Total Cost ($) | Cost per LYS | Illness Attack Rate (%) | LYS per 10,000 | Total Cost per person ($) | Cost per LYS |
| **Moderate Pandemics:**  **R = 1.9 and**  **CFR=0.25%** | Strategy B1: Vaccination-only | 6 months | 32.5 | 4* | $1,108 | - | 32.5 | 5* | $1,107 | - |
|  |  | 4 months | 32.5 | 5* | $1,106 | - | 32.4 | 6* | $1,103 | - |
|  |  | 2 months | 32.3 | 7* | $1,101 | - | 32.2 | 8* | $1,099 | - |
|  |  |  |  |  |  |  |  |  |  |  |
|  | Strategy B2: 2 months SD +AV+Vacc. | 6 months | 14.9 | 253 | $770 | $30,417 | 14.7 | 256 | $764 | $29,850 |
|  |  | 4 months | 13.6 | 272 | $732 | $26,850 | 13.0 | 281 | $715 | $25,472 |
|  |  | 2 months | 6.3 | 381 | $502 | $13,162 | 4.6 | 404 | $449 | $11,092 |
|  |  |  |  |  |  |  |  |  |  |  |
|  | Strategy B2: 4 months SD +AV+Vacc. | 6 months | 10.7 | 315 | $783 | $24,879 | 10.1 | 324 | $768 | $23,728 |
|  |  | 4 months | 5.4 | 394 | $581 | $14,728 | 4.6 | 405 | $555 | $13,693 |
|  |  | 2 months | 3.8 | 416 | $531 | $12,748 | 3.6 | 419 | $527 | $12,571 |
|  |  |  |  |  |  |  |  |  |  |  |
|  | Strategy B2: 6 months SD +AV+Vacc. | 6 months | 5.6 | 392 | $692 | $17,681 | 5.1 | 399 | $675 | $16,907 |
|  |  | 4 months | 4.6 | 406 | $658 | $16,212 | 4.3 | 409 | $652 | $15,941 |
|  |  | 2 months | 3.8 | 416 | $636 | $15,289 | 3.6 | 419 | $634 | $15,129 |
|  |  |  |  |  |  |  |  |  |  |  |
|  | Strategy B3: Sustained SD +AV+Vacc. | 6 months | 3.6 | 421 | $786 | $18,659 | 3.4 | 424 | $725 | $17,119 |
|  |  | 4 months | 3.1 | 428 | $758 | $17,734 | 2.9 | 430 | $588 | $13,670 |
|  |  | 2 months | 2.5 | 435 | $619 | $14,224 | 2.3 | 437 | $440 | $10,070 |

* indicates LYS value not statistically significantly different from zero due to stochastic simulation variation – Cost per LYS values with insignificant denominator have been omitted

**Table S2.3**: Vaccination delay and vaccination rate sensitivity analysis for extreme pandemics.

|  | | | **Vaccination Rate 1% per day** | | | | **Vaccination Rate 5% per day** | | | |
| --- | --- | --- | --- | --- | --- | --- | --- | --- | --- | --- |
| **Pandemic Scenarios** | **Mitigation Strategies** | **Delay in Vaccination** | **Illness Attack Rate (%)** | **LYS per 10,000** | **Total Cost ($)** | **Cost per LYS** | **Illness Attack Rate (%)** | **LYS per 10,000** | **Total Cost per person ($)** | **Cost per LYS** |
| **Extreme Pandemics:**  **R = 2.7 and**  **CFR = 1.5%** | Strategy C1: Vaccination-only | 6 months | 44.0 | 9* | $7,021 | - | 43.9 | 11* | $7,015 | - |
|  |  | 4 months | 43.9 | 13* | $7,012 | - | 43.9 | 15* | $7,006 | - |
|  |  | 2 months | 43.8 | 17* | $7,002 | - | 43.8 | 20* | $6,996 | - |
|  |  |  |  |  |  |  |  |  |  |  |
|  | Strategy C2:  2 months SD +AV+Vacc. | 6 months | 28.0 | 1219 | $4,797 | $39,340 | 27.9 | 1228 | $4,779 | $38,909 |
|  |  | 4 months | 27.6 | 1250 | $4,738 | $37,915 | 27.5 | 1257 | $4,723 | $37,575 |
|  |  | 2 months | 22.2 | 1737 | $3,828 | $22,043 | 17.4 | 2152 | $3,034 | $14,100 |
|  |  |  |  |  |  |  |  |  |  |  |
|  | Strategy C2:  4 months SD+AV+Vacc. | 6 months | 25.1 | 1458 | $4,477 | $30,704 | 24.6 | 1495 | $4,405 | $29,461 |
|  |  | 4 months | 16.1 | 2264 | $2,961 | $13,077 | 14.1 | 2432 | $2,636 | $10,839 |
|  |  | 2 months | 13.1 | 2511 | $2,482 | $9,884 | 12.9 | 2532 | $2,442 | $9,646 |
|  |  |  |  |  |  |  |  |  |  |  |
|  | Strategy C2:  6 months SD+AV+Vacc. | 6 months | 15.4 | 2329 | $2958 | $12,698 | 14.4 | 2409 | $2803 | $11,637 |
|  |  | 4 months | 13.7 | 2469 | $2689 | $10,890 | 13.5 | 2483 | $2659 | $10,711 |
|  |  | 2 months | 13.1 | 2512 | $2603 | $10,358 | 12.8 | 2532 | $2564 | $10,127 |
|  |  |  |  |  |  |  |  |  |  |  |
|  | Strategy C3: Sustained SD+AV+Vacc. | 6 months | 10.5 | 2720 | $2,332 | $8,571 | 10.4 | 2732 | $2,251 | $8,239 |
|  |  | 4 months | 10.0 | 2767 | $2,223 | $8,033 | 9.8 | 2781 | $2,031 | $7,304 |
|  |  | 2 months | 9.0 | 2839 | $1,959 | $6,899 | 8.9 | 2852 | $1,759 | $6,168 |
|  |  |  |  |  |  |  |  |  |  |  |
|  | Strategy C4: Sustained SD+WR+AV+Vacc. | 6 months | 6.9 | 3006 | $2,812 | $9,355 | 6.7 | 3019 | $2,612 | $8,654 |
|  |  | 4 months | 6.3 | 3054 | $2,669 | $8,738 | 6.1 | 3067 | $2,133 | $6,955 |
|  |  | 2 months | 5.4 | 3125 | $2,155 | $6,896 | 5.3 | 3133 | $1,601 | $5,111 |

* indicates LYS value not statistically significantly different from zero due to stochastic simulation variation – Cost per LYS values with insignificant denominator have been omitted

## Vaccination Coverage and Vaccine Efficacy

In order to gauge the maximum possible effect of vaccination, the baseline assumption for all scenarios considered in the main paper was that the vaccination campaign would achieve 100% coverage. Alternative coverage levels of 10% - 100% in 10% increments were examined. Figure S2.1 shows the final attack rate for different coverage levels, for strategies combining vaccination with antiviral measures and either limited or sustained duration social distancing.

The results show that when sustained social distancing strategies (red and green lines) are adopted, the full effect of vaccination can be achieved with less than 100% coverage. This is due to the herd immunity effect. The required level of vaccine coverage (30% to 50%) is somewhat lower than the required herd immunity coverage levels expected from a simple uniform mixing SIR epidemic model with R_0_ values of 1.8 and 2.5 (44% and 60% respectively). This is plausible, given that the simulated vaccination campaign preferentially targets those contributing most to transmission, and because by the time the vaccination campaign begins, some individuals will have become immune though infection, despite the operation of antiviral and social distancing interventions.

We have not further quantified the optimal vaccination coverage level, which would depend upon vaccination efficacy, vaccination rate, pandemic severity and transmissibility, details of non-vaccination interventions, vaccine development cost and per-unit vaccine production cost.

For limited duration social distancing measures (blue lines) where vaccination makes little impact (a 6 month vaccination delay was assumed), the level of coverage is irrelevant.


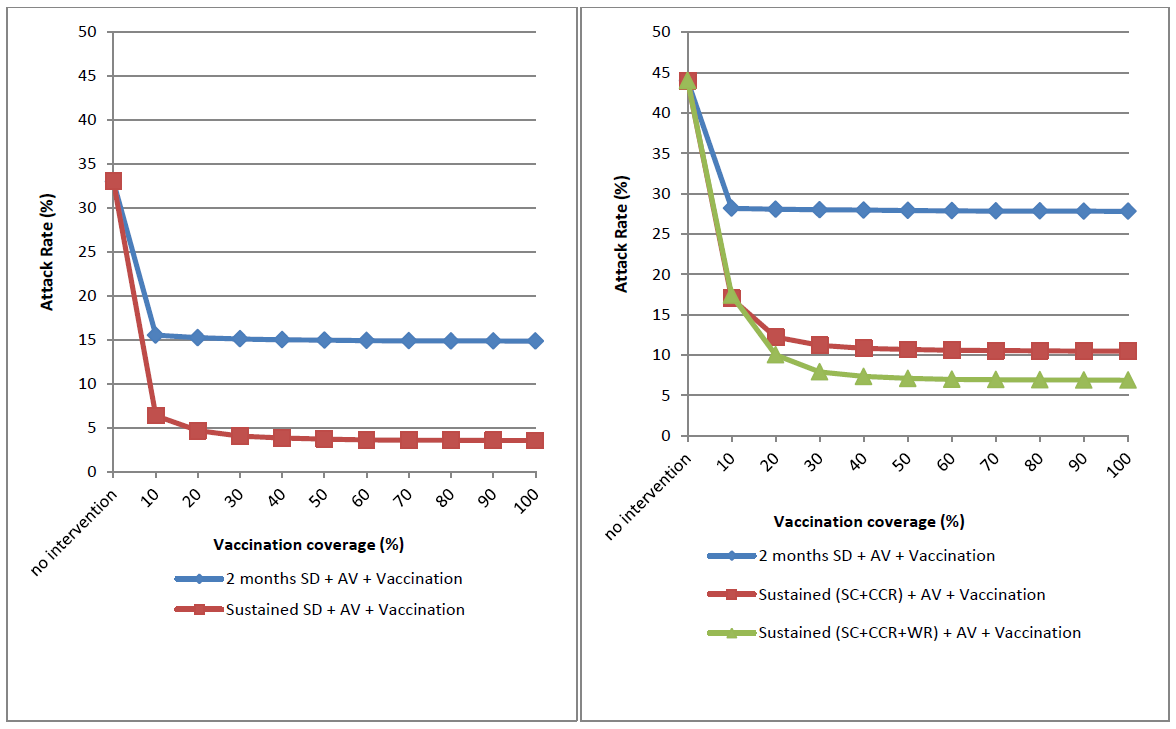


**Figure S2.1**: Final attack rates for vaccination strategies for two pandemic scenarios, assuming vaccination coverage levels from 10% to 100%, vaccination delay 6 months and vaccination rate of 1% per day.

An outcome of the vaccine efficacy sensitivity analysis was the observation that intervention strategies that assume 100% vaccination coverage are insensitive to vaccine efficacy parameter changes. All strategies had approximately identical effectiveness and cost outcomes for vaccine efficacy values in the range of 65% - 85% (see Table S2.4). Given that the vaccination coverage analysis (see above) showed that the full effect of vaccination could be achieved with approximately 50% coverage of a 75% effective vaccine, it is not surprising that 100% coverage of a 65% vaccine would be equally effective.

## Table S2.4: Vaccine efficacy sensitivity analysis

|  |  | **Vaccine Efficacy 85%** | | | | **Vaccine Efficacy 65%** | | | |
| --- | --- | --- | --- | --- | --- | --- | --- | --- | --- |
| **Pandemic Scenarios** | **Mitigation Strategies** | **Illness Attack Rate (%)** | **LYS per 10,000** | **Total Cost per person ($)** | **Cost per LYS** | **Illness Attack Rate (%)** | **LYS per 10,000** | **Total Cost per person ($)** | **Cost per LYS** |
| **Mild pandemics** | Strategy A1 | 13.5 | 1 | $209 | $1,407,390 | 13.5 | 1* | $209 | - |
|  | Strategy A2 | 3.8 | 19 | $178 | $92,704 | 3.9 | 19 | $178 | $93,926 |
|  | Strategy A3 | 3.3 | 20 | $178 | $88,822 | 3.4 | 20 | $179 | $89,988 |
|  |  |  |  |  |  |  |  |  |  |
| **Moderate pandemics** | Strategy B1 | 32.6 | 2 | $589 | $3,489,658 | 32.6 | 2 | $590 | $3,825,046 |
|  | Strategy B2 | 14.9 | 102 | $529 | $51,624 | 15.0 | 102 | $530 | $52,047 |
|  | Strategy B3 | 3.5 | 170 | $732 | $42,945 | 3.6 | 170 | $733 | $43,206 |
|  |  |  |  |  |  |  |  |  |  |
| **Extreme pandemics** | Strategy C1 | 44.0 | 10 | $7,018 | $7,072,946 | 44.0 | 8 | $7,023 | $8,720,427 |
|  | Strategy C2 | 28.0 | 1220 | $4,796 | $39,312 | 28.0 | 1217 | $4,801 | $39,437 |
|  | Strategy C3 | 10.5 | 2726 | $2,322 | $8,520 | 10.7 | 2712 | $2,348 | $8,658 |
|  | Strategy C4 | 6.9 | 3010 | $2,804 | $9,317 | 7.0 | 2998 | $2,828 | $9,433 |

* indicates LYS value not statistically significantly different from zero due to stochastic simulation variation – Cost per LYS values with insignificant denominator have been omitted

Strategies are abbreviated as tables S2.1, S2.2 and S2.3 .

## Vaccination Coverage and Vaccine Efficacy

In the main results it was assumed that individual susceptibility to infection differed by age, resulting in age-specific infection rates similar to the 2009 pandemic, where 18-24 years olds had the highest attack rates while those 25 years and older had the lowest [[1](#_ENREF_1)]. Previous pandemics have exhibited different age-specific attack rate profiles. The 1957 pandemic resembled seasonal influenza with the highest attack rates in children, while the 1968 pandemic had similar attack rates in all age groups [[2](#_ENREF_2)]. The sensitivity of the results to an alternative assumption that all age groups would be equally susceptible was examined.

The result was a shift in the burden of illness to older age groups, and as a result, slightly fewer (less than 12%) life years were saved by interventions. However the shift of illness to older age groups also reduced the death-related productivity losses, resulting in lower total pandemic costs and slightly improved cost effectiveness of interventions.

## Table S2.5 : Sensitivity of effectiveness and cost effectiveness to age-specific susceptibility

|  | **2009 H1N1 Age Specific Susceptibility** | | | | **Flat Age Specific Susceptibility** | | | |
| --- | --- | --- | --- | --- | --- | --- | --- | --- |
| **Mitigation Strategies** | **Attack Rate (%)** | **Life Years Saved per 10000** | **Total Cost ($) per person** | **Cost ($) per LYS** | **Attack Rate (%)** | **Life Years Saved per 10000** | **Total Cost ($) per person** | **Cost ($) per LYS** |
| **Mild Pandemics (R=1.5 and CFR=0.03%)** | | | | | | | | |
| **No intervention** | 14 | - | $170 | $0 | 15 | - | $169 | $0 |
| **Strategy A3: 8 weeks of SD + AV + Vaccination** | 3 | 20 | $179 | $89,574 | 3 | 20 | $175 | $85,729 |
| **Moderate Pandemics (R=1.9 and CFR=0.25%)** | | | | | | | | |
| **No intervention** | 33 | - | $1,031 | $0 | 33 | - | $992 | $0 |
| **Strategy B3: Sustained SD + AV + Vaccination** | 4 | 421 | $786 | $18,659 | 4 | 377 | $682 | $18,093 |
| **Extreme Pandemics (R=2.7 and CFR=1.5%)** | | | | | | | | |
| **No intervention** | 44 | - | $6,953 | $0 | 45 | - | $6,767 | $0 |
| **Strategy C3: Sustained SD + AV + Vaccination** | 11 | 2720 | $2,332 | $8,571 | 9 | 2676 | $2,026 | $7,571 |

Pandemic outcomes are given for two different age-specific susceptibility to infection profiles. On the left (unshaded) the age-specific susceptibility gives a 2009-like age-specific attack rate; on the right (shaded) all age groups are equally susceptible. Outcomes reported (columns) are symptomatic attack rate, life years saved (LYS) per 10,000 population, total pandemic cost per person, and cost per LYS. For each pandemic scenario (mild, moderate and extreme), outcomes are reported for the no-intervention strategy, and strategy consisting of rigorous social distancing (duration 8 weeks for mild scenario, sustained for moderate and extreme), antiviral treatment and prophylaxis, and vaccination after 6 months.

# Additional Results

## Incremental Cost Effectiveness Ratios

**Table S2.6**: Incremental cost effectiveness ratios

|  |  |  | **Incremental Cost Effectiveness Ratio**  **$ per LYS** | | | |
| --- | --- | --- | --- | --- | --- | --- |
| **strategy** | **Total Cost ($) per person** | **LYS per person** | **strategy vs**  **no intervention** | **strategy vs**  **no vaccination** | **sustained SD vs**  **8 weeks** | **8 weeks vs**  **2 weeks SD** |
|  |  |  |  |  |  |  |
| **A1** | 210 | 0.000141206 | 283273 | 283273 |  |  |
| **A1'** | 170 | 0 |  |  |  |  |
| **A2** | 178 | 0.002126999 | 3761 | 60245 |  |  |
| **A2'** | 141 | 0.001512843 | -19169 |  |  |  |
| **A3** | 179 | 0.001998348 | 4504 | 147694 |  | -7773 |
| **A3'** | 143 | 0.001754601 | -15388 |  |  | 8273 |
|  |  |  |  |  |  |  |
| **B1** | 1108 | 0.000413704 | 186123 | 186123 |  |  |
| **B1'** | 1031 | 0 |  |  |  |  |
| **B2** | 770 | 0.025314791 | -10310 | 28947 |  |  |
| **B2'** | 719 | 0.02355292 | -13247 |  |  |  |
| **B3** | 786 | 0.042124444 | -5816 | -9090 | 952 |  |
| **B3'** | 858 | 0.034203707 | -5058 |  | 13051 |  |
|  |  |  |  |  |  |  |
| **C1** | 7021 | 0.00086623 | 78501 | 78501 |  |  |
| **C1'** | 6953 | 0 |  |  |  |  |
| **C2** | 4797 | 0.12193696 | -17681 | 696 |  |  |
| **C2'** | 4794 | 0.117623966 | -18355 |  |  |  |
| **C3** | 2332 | 0.272080271 | -16984 | -17748 | -16418 |  |
| **C3'** | 4169 | 0.168573855 | -16515 |  | -12267 |  |
| **C4** | 2812 | 0.328083071 | -12622 | -22034 |  |  |
| **C4'** | 5310 | 0.214710283 |  |  |  | -7652 |

Incremental cost effectiveness ratios (ICER) are given in $ per LYS for each strategy compared to no interventions, and between strategies that differ only one intervention element. Strategies are abbreviated as tables S2.1, S2.2 and S2.3. Negative values indicate that the strategy (or added strategy element) reduces both mortality and total cost.

# References

1. ECDC: **ECDC Risk Assessment 2009 influenza A(H1N1) pandemic Version 7**. In: *ECDC Risk Assessemnt.* Stockholm: European Center for Disease Control; 2009.

2. Glezen WP: **Emerging infections: pandemic influenza**. *Epidemiol Rev* 1996, **18**(1):64-76.
